# Echocardiography Parameters and Self-Care Among Patients With Heart Failure

**DOI:** 10.7759/cureus.102561

**Published:** 2026-01-29

**Authors:** Maria Zakka, Victoria Alikari, Dimos Mastrogiannis, Angeliki Stamou, Eirini Nikou, Eftychia Chamodraka, Maria Polikandrioti

**Affiliations:** 1 Department of Nursing, Postgraduate Program "Applied Clinical Nursing", University of West Attica, Athens, GRC; 2 Department of Cardiology, Asklepieion Voulas General Hospital, Athens, GRC

**Keywords:** echocardiography parameters, heart failure, inferior vena cava, left ventricular ejection fraction, self-care behaviors

## Abstract

Introduction: The clinical syndrome of heart failure (HF) is associated with high morbidity and frequent hospitalizations. Echocardiography, widely known as cardiac ultrasound, is a noninvasive diagnostic method that provides detailed information on cardiac structure and function. Therefore, the rationale for this study was that objective echocardiography parameters may reflect the clinical status and severity of symptoms which in turn help identify HF patients at risk for poor self-care. The aim of the present study was to explore which echocardiographic parameters are associated with self-care among HF patients.

Methods: In this cross-sectional study, 130 HF outpatients were enrolled. Data collection was performed by the completion of the European Heart Failure Self-care Behaviour Scale (EHFScBS). Also, patients' echocardiography parameters were recorded. Participants were selected using the method of convenience sampling. Statistical significance was set at 0.05%.

Results: The mean self-care score, as measured by the EHFScBS, was 25.5±7.3 (range 9-45), indicating moderate levels of self-care. The EHFScBS score was weakly but statistically significantly positively correlated with left ventricular ejection fraction (LVEF) (rho=0.199; p=0.029), indicating that higher LVEF values were associated with better self-care. On the contrary, the EHFScBS score was weakly but significantly negatively correlated with the early diastolic transmitral flow velocity-to-early diastolic mitral annular velocity ratio (E/e' ratio) (rho=-0.290; p=0.001), inferior vena cava (IVC) (rho=-0.289; p=0.001), and left atrium (LA) diameter (rho=-0.305; p=0.001), suggesting that higher values of these indicators were associated with poorer patients' self-care.

Conclusions: An improved understanding of the link between echocardiographic factors and self-care can help healthcare providers develop targeted interventions and support programs to improve self-care.

## Introduction

The progressive and unpredictable clinical syndrome of heart failure (HF) is an important public health issue that affects approximately 15 million people in Europe, 6.5 million in the United States, and more than 37.7 million worldwide. HF prevalence exceeds 12% in adults aged 80 years and older and is higher in countries where the elderly predominate [1]. Furthermore, the age-standardized prevalence in the Eastern Mediterranean region is 706.43 per 100,000 persons [2].

HF management incorporates pharmacological and non-pharmacological interventions, as well as implanted cardiac devices. In the non-pharmacological context, self-care plays a vital role in preventing decompensation, re-hospitalization, and frequent use of healthcare services [1,3]. Self-care involves daily tasks such as monitoring body weight, staying physically active, following dietary limitations, including salt restriction, and prompt recognition of the symptoms' deterioration [3]. Self-care theories in HF, such as the Situation-Specific Theory of Heart Failure Self-Care, conceptualize self-care as a dynamic process influenced by symptom perception, functional capacity, decision-making ability, confidence, prior experience, and abilities of each individual personal and environmental determinants [3]. Moreover, social factors such as psychological stress and absence of family support determine clinical outcomes. Therefore, social determinants in combination with biological risk create the HF patients' profile [4], which in turn may affect self-care. Echocardiographic parameters provide objective markers of cardiac burden [5]. Within the theoretical framework of self-care, higher echocardiographic burden may indirectly influence self-care through increased symptom severity and functional limitation rather than through a direct behavioral pathway.

Echocardiography provides accurate information about cardiac function during its performance [5-7]. More in detail, this noninvasive and widely used procedure quantifies the left ventricular ejection fraction (LVEF) and evaluates several other markers for assessing congestion, such as diastolic function, filling pressures, chamber dimensions, types of valvular heart diseases, and the diameter of the inferior vena cava (IVC) [5]. Abnormal values of the aforementioned indices are predictors of one-year mortality and hospital admission in HF patients with preserved LVEF (>50%) [6]. In patients with HF and preserved LVEF, an analysis of 46 studies including 20,056 patients demonstrated that reduced left ventricular global longitudinal strain, left atrial reservoir strain, and the ratio of tricuspid annular plane systolic excursion to pulmonary artery systolic pressure were consistently associated with increased mortality and hospitalization [8]. Also, high rates of mortality and readmissions were observed in HF patients with reduced LVEF <25% and pulmonary artery systolic pressure >40 mmHg [7]. Echocardiography is a valuable tool in the diagnosis and management of HF patients since it determines systolic or diastolic dysfunction, provides information on pathophysiology and hemodynamics, and may help identify patients with a poor prognosis [5-7].

However, echocardiography indices are not a direct indicator of self-care among HF patients but may reflect patients' symptoms and quality of life (QoL), thus providing clinicians with an explanatory frame between cardiac function and self-care [5]. Moreover, the association between echocardiographic parameters and self-care behaviors may be particularly relevant in HF with reduced ejection fraction (HFrEF). In HF, the reduced LVEF is a key marker of disease severity closely linked to symptom burden and self-care capacity. Although primarily focused on systolic dysfunction, other echocardiographic parameters such as left atrial enlargement also provide important information on diastolic function and filling pressures, which may further influence self-care behaviors in this population.

The incorporation of echocardiography markers into routine clinical evaluation in conjunction with the assessment of self-care may improve clinical outcomes by facilitating the early identification of high-risk individuals for adverse events. After HF diagnosis, this combined approach may help clinicians to guide more effectively personalized treatment and facilitate targeted follow-up strategies.

To the best of our knowledge, evidence on the association between self-care and echocardiographic parameters are limited in existing studies. The aim of the present study was to explore echocardiographic parameters linked to self-care among HF patients.

## Materials and methods

Study design and participants

In this cross-sectional study, 130 community-dwelling patients with chronic HF, who attended the outpatient department of Asklepieion Voulas General Hospital for scheduled follow-up, were enrolled.** **The study was carried out from December 2024 to June 2025. The sample was selected using convenience sampling. 

Inclusion and exclusion criteria of the sample

The inclusion criteria were (i) the ability to write, read, and comprehend the Greek language, (ii) the ability to read and sign the informed consent form, and (iii) patients with compromised LVEF. 

Exclusion criteria included patients who (i) were hospitalized, (ii) had mental disorders (anxiety, depression), or (iii) had cognitive impairment. Mental disorders and cognitive impairment were assessed by the cardiologist. 

Data collection

The echocardiographic parameters were assessed by the cardiologist using cardiac ultrasound (Simpson's method), while the other variables were collected through the interview method after the end of follow-up. Echocardiographic evaluations were conducted the same day immediately after the self-care assessments to ensure that cardiac function measurements corresponded with the patients' reported self-care behaviors.

Research instrument

Data were collected using the European Heart Failure Self-care Behaviour Scale (EHFScBS) (Appendices), along with patients' characteristics.

HF patients' characteristics

The characteristics of HF patients included gender, age, and New York Heart Association (NYHA) class. According to the classification of NYHA, stage I indicates no limitations with ordinary activity, stage II slight limitations with symptoms during moderate activity, stage III marked limitations with symptoms during minimal activity, and stage IV severe symptoms at rest or with any activity.

The echocardiography parameters included in this study were the following: LVEF, early-to-atrial diastolic transmitral flow velocity ratio (E/A ratio), early diastolic transmitral flow velocity-to-early diastolic mitral annular velocity ratio (E/e′ ratio), IVC diameter, left ventricular end-diastolic diameter (LVEDD), and left atrium (LA) diameter. 

In ultrasound, the normal values ​​of the echocardiography parameters are as follows: E/A ratio: up to 1.2 m/s; E/e′ ratio: up to 8 cm/s; IVC diameter: ≤21 mm; LVEDD: 35-56 mm; and LA diameter: 10-39 mm.

These echocardiographic parameters were selected because they are routinely obtained during echocardiography, are well validated, and have established clinical relevance in the assessment of cardiac structure and function in HF. LVEF and LVEDD reflect left ventricular systolic performance and remodeling, while the E/A and E/e′ ratios reflect the left ventricular diastolic function and filling pressures. LA diameter serves as a marker of chronic diastolic burden, and IVC diameter estimates volume status and right atrial pressure. The measurement of echocardiographic parameters took place only once. There was no measurement of two or three times, and the average value of these measurements was not recorded. Both NYHA class and echocardiographic parameters were assigned at the same visit. 

Also, the participants' self-reports were recorded regarding QoL, dyspnea, and changes in daily activities within a range of values from 0 to 10. 

EHFScBS

For the evaluation of self-care in HF patients, EHFScBS was used. It initially included 12 items, while the latter included nine items (nine-item version, EHFScB-9). This widely used scale has been controlled for reliability and validity in several languages [9], including Greek [10]. The EHFScB-9 scale includes items addressing self-care, such as daily weighing, adherence to medication, and seeking medical help at the worsening of symptoms. Each item of the EHFScB-9 scale rates on a 5-point Likert scale (1=completely agree to 5=completely disagree), while the total score of the scale ranges from 9 to 45. Lower scores indicate better self-care. Cronbach's alpha (α) of the EHFScB-9 scale is 0.80, demonstrating good internal consistency. The scale was validated for the Greek population by Lambrinou et al. [10]. The scale is freely available on the internet (https://liu.se/en/research/european-heart-failure-self-care-behaviour-scale). 

Ethical considerations

This study was approved by the Ethics Committee of Asklepieion Voulas General Hospital (approval number: 308/07.11.2024). All HF patients participated in this study after providing written informed consent. Also, they were informed about their right to refuse participation or withdraw from the study, in accordance with the ethical principles of the Declaration of Helsinki (1989) by the World Medical Association. Anonymity and confidentiality were ensured at data collection.

Statistical analysis

Qualitative data are presented as absolute (n) and percentage (%) frequencies, while quantitative data are presented with the mean, standard deviation (SD), median, and interquartile range (IQR). The normality of quantitative measures was assessed using the Kolmogorov-Smirnov test and graphically through histograms and Q-Q plots. The linear correlation between self-care scores and patients' quantitative characteristics was assessed using Spearman's rho, within a range of values from -1 to +1. Values close to -1 or +1 indicate a strong negative or positive linear correlation, respectively, while values close to 0 indicate the absence of linear correlation. Multiple linear regression was performed to assess the effect of potential factors on self-care scale scores. Results are presented with β regression coefficients and 95% confidence intervals (CI). A p-value of <0.05 was considered statistically significant. All statistical analyses were conducted using IBM SPSS Statistics for Windows, Version 28.0 (IBM Corp., Armonk, New York, United States).

## Results

Descriptive results are presented in Table 1. The majority of participants were male (65.4%), aged 71-80 years (40%), and in NYHA class III (46.1%).

**Table 1:** Description of the sample of HF patients (n=130) NYHA: New York Heart Association; HF: heart failure

Patients' demographic and clinical characteristics	n (%)
Gender	
Male	85 (65.4%)
Female	45 (34.6%)
Age (years)	
≤60	10 (7.7%)
61-70	25 (19.2%)
71-80	52 (40%)
>80	43 (33.1%)
NYHA class	
I	8 (6.2%)
II	32 (24.6%)
III	60 (46.1%)
IV	30 (23.1%)

Regarding the patients' quantitative clinical measurements (Table 2), the mean LVEF was 38.1%, while the mean E/A and E/e' ratios were 1.2 and 10.9, respectively. The mean values of the echocardiographic measurements IVC, LVEDD, and LA diameter were 18 mm, 55.6 mm, and 51.7 mm, respectively. In terms of participants' self-reports in a range of 0-10, the median QoL score was 5 (moderate level), the median dyspnea score was 2 (low level), and the score for changes in daily activities was 6 (indicating a moderate level).

**Table 2:** Description of the quantitative measurements of HF patients (n=130) SD: standard deviation; IQR: interquartile range; LVEF: left ventricular ejection fraction; E/A ratio: early-to-atrial diastolic transmitral flow velocity ratio; E/e' ratio: early diastolic transmitral flow velocity-to-early diastolic mitral annular velocity ratio; IVC: inferior vena cava; LVEDD: left ventricular end-diastolic diameter; LA diameter: left atrium diameter; HF: heart failure

Echocardiographic parameters and self-reported measures	Mean(±SD)	Median (IQR)
LVEF	38.1(±9.5)	38.0 (30.0-45.0)
E/A ratio	1.2(±0.8)	0.9 (0.7-1.5)
E/e' ratio	10.9(±4.9)	10.0 (8.0-13.0)
IVC (mm)	18.0(±4.8)	18.0 (15.0-20.0)
LVEDD (mm)	55.6(±8.0)	55.0 (50.0-61.0)
LA diameter (mm)	51.7(±8.9)	50.5 (45.5-56.0)
Quality of life (score range: 0-10)	5.3(±2.3)	5.0 (4.0-7.0)
Dyspnea after (score range: 0-10)	3.0(±3.0)	2.0 (0.0-5.0)
Changes in activities (score range: 0-10)	6.0(±2.9)	7.0 (5.0-8.0)

Patients' self-care

Table 3 presents the results from the self-care questionnaire. The mean self-care score, as measured by the EHFScBS, was 25.5±7.3 (range 9-45), and the median (IQR) was 25.0 (20.0-31.0). This score indicates a moderate level of self-care.

**Table 3:** Description of self-care of HF patients (n=130) SD: standard deviation; IQR: interquartile range; HF: heart failure

	Mean(±SD)	Median (IQR)
European Heart Failure Self-care Behaviour Scale (range 9-45)	25.5(±7.3)	25.0 (20.0-31.0)

Factors associated with patient self-care

Table 4 presents the association between patients' characteristics (echocardiography parameters, self-reports) and their self-care. Statistically significant associations were observed between the EHFScBS score and LVEF (p=0.029), E/e' ratio (p=0.001), IVC (p=0.001), LA diameter (p=0.001), and the self-rated items: QoL (p=0.035), dyspnea (p=0.001), and the change in daily activities score (p=0.001). More specifically, the EHFScBS was found to be statistically significantly positively and weakly correlated with LVEF (rho=0.199; p=0.029) and self-rate of QoL (rho=0.192; p=0.035). The greater the LVEF and QoL score, the better the patients' self-care. On the contrary, the EHFScBS was significantly and negatively weakly correlated with the E/e' ratio (rho=-0.290; p=0.001), IVC (rho=-0.289; p=0.001), LA diameter (rho=-0.305; p=0.001), as well as self-rates of dyspnea (rho=-0.341; p=0.001), and change in activities (rho=-0.289; p=0.001). The higher these indicators, the worse the patients' self-care.

**Table 4:** Association between echocardiography parameters, self-reports, and self-care (n=130) *Statistical significance (p<0.05) CI: confidence interval; LVEF: left ventricular ejection fraction; E/A ratio: early-to-atrial diastolic transmitral flow velocity ratio; E/e' ratio: early diastolic transmitral flow velocity-to-early diastolic mitral annular velocity ratio; IVC: inferior vena cava; LVEDD: left ventricular end-diastolic diameter; LA diameter: left atrium diameter; QoL: quality of life

	Spearman's rho	P-value	95% CI
LVEF	0.199	0.029*	0.015-0.370
E/A ratio	-0.115	0.213	-0.293-0.071
E/e' ratio	-0.290	0.001*	-0.450 to -0.111
IVC	-0.289	0.001*	-0.449 to -0.110
LVEDD	-0.132	0.151	-0.309-0.054
LA diameter	-0.305	0.001*	-0.463 to -0.127
Self-rated QoL (score range: 0-10)	0.192	0.035*	0.008-0.364
Self-rated dyspnea (score range: 0-10)	-0.341	0.001*	-0.494 to -0.167
Self-rated change in activities (score range: 0-10)	-0.289	0.001*	-0.450 to -0.111

Assessment of the impact of factors on patient self-care 

Multiple linear regression, with the enter method, was subsequently performed to assess the impact of the characteristics that showed statistically significant associations with patient self-care. The results are presented in Table 5. The adjusted R^2^ of the model reached 22.3%. The mean variance inflation factor (VIF) value was 1.6, indicating no multicollinearity effect. 

**Table 5:** The impact of echocardiographic characteristics on patients' self-care *Statistical significance (p<0.05) CI: confidence interval; LVEF: left ventricular ejection fraction; E/A ratio: early-to-atrial diastolic transmitral flow velocity ratio; E/e' ratio: early diastolic transmitral flow velocity-to-early diastolic mitral annular velocity ratio; IVC: inferior vena cava; LVEDD: left ventricular end-diastolic diameter; LA diameter: left atrium diameter; QoL: quality of life

Echocardiographic parameters and self-reported measures	β coefficient (95% CI)	P-value
LVEF	0.06 (-0.08-0.19)	0.421
E/e' ratio	0.09 (-0.17-0.35)	0.490
IVC	-0.36 (-0.67 to -0.05)	0.025*
LVEDD	-	-
LA diameter	0.05 (-0.12-0.22)	0.541
Self-rated QoL	-0.78 (-1.48 to -0.08)	0.029*
Self-rated dyspnea	0.07 (-0.50-0.64)	0.811
Self-rated change in activities	-0.51 (-1.11-0.10)	0.098

It was found that an increase of one unit in the IVC index and the QoL score was associated with a decrease in self-care scores on the EHFScBS by 0.36 and 0.78 points, respectively (β=-0.36; 95% CI: -0.67 to -0.05; p=0.025 and β=-0.78; 95% CI: -1.48 to -0.08; p=0.029, respectively).

## Discussion

According to the results, the mean score on the EHFScBS was 25.5±7.3, indicating moderate self-care behaviors. In a study by D'Souza et al., among 160 HF patients (75% of whom were men, with a mean age of 56.1±7.9 years and 60.6% presenting with LVEF <30%), the mean self-care behavior score was 45.0±12.4 out of 100, as measured with the EHFScBS-9 [2]. Similarly, in a review by Sedlar et al., it was reported that age, sex, education, NYHA class, QoL, and depressive symptoms were the factors most frequently associated with EHFScBS scores. Also, the review identified LVEF as one of the factors most commonly correlated with self-care behaviors as measured by the EHFScBS [11]. However, it is crucial not only to document self-care scores but also to identify strategies to improve them.

In the present study, the echocardiography parameters assessed were LVEF, E/A ratio, E/e′ ratio, IVC diameter, LVEDD, and LA diameter. These features may reflect patients' functional status and engagement in self-care behaviors, while abnormal values exert a negative impact on illness perception. Moreover, LVEF values could serve as a clinical indicator relevant to self-care assessment for health professionals [10-12].

LVEF reflects global systolic performance, while the E/A and E/e′ ratios serve as indicators of diastolic function and left ventricular filling pressure, which may substantially influence symptom burden and functional capacity. IVC diameter provides an estimate of right atrial pressure and intravascular volume status, while LVEDD reflects ventricular remodeling that may affect exercise tolerance and daily activity. Finally, LA diameter reflects longitudinal atrial function and is related to cardiac performance during systole [6-8].

LVEF is the most commonly measured echocardiographic index and reflects the systolic function of the left ventricle. It serves as a primary diagnostic parameter for HF or a marker of disease severity [11] and has been associated with self-care in HF patients. Lycholip et al., using the same scale (EHFScBS) in a sample of 118 HF participants (mean age: 69±11.5 years; 70% male), demonstrated that a lower LVEF (per 5% decrease) was associated with worsening self-care behavior [13]. Similarly, Pobrotyn et al., in a sample of 403 patients with chronic HF (mean LVEF 40.53%), illustrated that an increase in LVEF% was a significant positive predictor of self-care scores [14]. On the other end of the spectrum, Kim et al. supported that illness perception and social support indirectly influence LVEF through the mediating effects of self-efficacy and self-care compliance [12]. Low LVEF values reflect symptom burden, limited physical ability, and disease severity or progression (fatigue, dyspnea, and exercise intolerance). These parameters impose limitations on performing self-care activities, such as monitoring weight, managing fluid intake, and recognizing early signs of decompensation. In turn, these limitations adversely impact patients' perception of their illness [12-14]. It is worth emphasizing that in the present study, EHFScBS was statistically significant and positively correlated with LVEF, although the strength of this association was weak. A possible explanation is that LVEF is predominantly determined by disease severity and the underlying pathophysiology, including myocardial contractility and reverse structural remodeling, rather than solely by patient-reported self-care behaviors. In clinical care, this finding illustrates the importance of an approach that combines subjective assessments (self-care) with objective ones (echocardiographic indices) as they provide valuable complementary information for HF management.

A possible explanation for the finding that higher IVC values are associated with poorer patients' self-care may be attributed to non-adherence to dietary restrictions and the therapeutic regimen. In HF, congestion mainly arises from either high salt intake (which leads to water retention and increased blood volume) or poor medication adherence to diuretics (reduced fluid removal). Both mechanisms increase volume and right-sided pressures, which in turn increase the IVC values. For instance, Tchernodrinski et al. [15] among 70 HF hospitalized acute decompensated HF patients receiving intravenous furosemide showed a median baseline IVC diameter of 2.38 cm (interquartile range: 1.91-2.55 cm). One to two hours after receiving furosemide, their IVC diameter decreased by an average of 0.21 cm and remained significantly lower than baseline at 2-3 hours post-treatment, with a 0.15 cm average reduction. IVC within normal range is essential for optimal volume status and reduced heart strain. This noticeable finding may guide interventions for volume management in HF [15,16]. The IVC diameter is an index of central venous pressure. Therefore, fluid overload is reflected by a dilated, non-collapsible IVC, as measured in echocardiography. A dilated and low collapsibility IVC is associated with higher readmission rates and increased mortality risk among acutely decompensated hospitalized HF patients [17,18] and, more in detail, in HF patients with NYHA classes I-IV independently predicts rehospitalization after up to one year [19,20]. Furthermore, a dilated IVC (>21 mm) is a predictor of renal function worsening in patients with advanced decompensated HF [16].

The echocardiographic measure of diastolic function and filling pressure, the E/e′ ratio, was also associated with EHFScBS scores. Patients with higher E/e′ values, indicating impaired diastolic function and elevated filling pressures, tended to exhibit worse self-care behaviors. More specifically, self-care behaviors can influence cardiac stress and filling pressures, which are reflected in the E/e′ ratio; better self-care may help maintain lower filling pressures and support overall cardiac function [21]. This association suggests a link between physiological status (heart function) and self-care behavior, highlighting that effective HF management may positively influence measurable cardiac function.

One hypothesis that was not demonstrated in this study was that self-care behaviors (medication adherence, dietary and fluid management) may influence ventricular remodeling and thereby indirectly affect LVEDD over time. In the present study, LVEDD was not associated with self-care behaviors. An explanation for this finding is that it ranged within normal values (mean 55.6 mm within a range of scores 35-56 mm). Structural remodeling reflected by LVEDD may be present without severe symptoms and may influence patients' motivation or ability to engage in self-care. Further research specifically examining the relationship between LVEDD and self-care behaviors would be valuable. However, LVEDD is known to be linked to cardiovascular events and all-cause mortality [22]. 

The LA diameter was found to be associated with self-care in this study. This index refers to the structural remodeling of the LA and is influenced by the chronic elevation of left ventricular filling pressures. Reduced LVEF reflects impaired left ventricular systolic performance, resulting in increased left atrial workload to preserve ventricular filling. When the LA diameter is low, the atrium can no longer sustain the compensatory mechanism. As an indicator of LA function, LA diameter may predict adverse cardiovascular outcomes [23]. Lower LA diameter values may therefore be linked to poorer heart function and limited functional capacity, which in turn could negatively affect patients' ability to perform self-care behaviors.

An important aspect not included in the present study, but important to note, is that the LA strain offers insights into atrial stiffness, elevated left ventricular filling pressures, and early diastolic dysfunction. LA strain has emerged as a valuable measure for the assessment of left ventricular diastolic dysfunction [24].

According to the results, the higher the dyspnea and the greater the change in activities, the worse the patients' self-care. Indeed, severe symptoms and functional impairment are associated with poor self-care. This can lead to higher E/e′ ratios, elevated filling pressures, and a potential decline in LVEF over time. Dyspnea is a burdensome symptom affecting QoL in both ambulatory and inpatient HF patients. Notably, dyspnea is the leading cause of emergency healthcare utilization [25] and persists at hospital discharge in approximately 25% of HF patients [26]. Persistent dyspnea is associated with diminished QoL and frequent hospital readmissions [27]. Paroxysmal nocturnal dyspnea has also been associated with perceived social isolation [28].

Dyspnea physically limits activity, reduces energy, and affects mood, all of which make adherence to self-care behaviors more challenging for patients, thereby creating a cycle that further compromises HF outcomes [25-27].

There is a strong, bidirectional association between self-care and how HF patients evaluate their QoL. Better self-care tends to improve QoL, while poor QoL, in turn, may undermine self-care behaviors. Patients' perceptions of their illness may affect care behaviors [29]. Undoubtedly, interventions aimed at enhancing self-care are important for the successful management of HF. However, no effort will bring the anticipated result unless patients' attitudes towards the disease and its treatment are first explored [30].

Multiple linear regression analysis revealed that increased IVC index and worse QoL scores were independently associated with poorer self-care as measured by the EHFScBS. Specifically, for each one-unit increase in the IVC index, the self-care score decreased by 0.36 points, and for each one-unit increase in the QoL score, the self-care score decreased by 0.78 points. These results indicate that greater volume overload (as reflected by IVC) and poorer QoL independently predict worse self-care behaviors in HF patients. Of note, the cross-sectional design of this study limits the ability to determine the directionality or causality of the observed associations between self-care behaviors and echocardiographic indices. While increased IVC index and poorer QoL were independently associated with worse self-care, it remains unclear whether deteriorating cardiac hemodynamics lead to reduced self-care or, conversely, whether inadequate self-care contributes to worsening cardiac status. Longitudinal studies are needed to clarify these temporal relationships and better understand how changes in cardiac structure and function influence, or are influenced by, self-care behaviors over time.

Echocardiography is the key to diagnosing heart conditions with portable handheld ultrasound devices to provide image quality at the bedside. Possibly, these simple and affordable devices may help with prompt decision-making [31] when self-care is inadequate. Furthermore, remote monitoring of worsening indicators and early intervention helps prevent emergency hospital admissions. Through telemonitoring, traditional care with scheduled visits shifts to an effective management approach integrated into the patient's daily life. Regular measurements encourage patients' active involvement in their care. Even during stable periods, data from telemonitoring systems offer a comprehensive view of the patient's condition, enhancing clinical decision quality [32].

This study explored the relationship between echocardiographic parameters and patients' self-care using the EHFScBS and showed that objective measurements are related to self-care. Interestingly, a study conducted by Smith et al. [33] supports that clinical scientists typically do not share echocardiography results directly with patients. Instead, they prepare a report for the requesting clinician, who then discusses the findings with the patient. In their pilot study conducted in an echocardiography outpatient department, 19 out of 20 patients expressed a preference to receive their results directly from the clinical scientist immediately after the echocardiogram, rather than waiting to discuss them with their doctor at a later appointment. This prompt communication may enhance patients' engagement in self-care by increasing their awareness of their health status. The uniqueness of this study lies in its focus on the relationship between objective echocardiographic measurements and patients' self-care and furthermore establishes the basis for integrating patients' preferences for receiving immediate feedback.

In this study, the EHFScBS score showed a weak but statistically significant positive correlation with LVEF and weak but significant negative correlations with the E/e' ratio, IVC, and LA. These findings likely reflect the complex interactions between clinical status and cardiac function, variability in symptom perception and motivation, diverse physical and educational factors, and the study's sample size. In the progressive clinical syndrome of HF, where multiple factors influence patient outcomes, even weak correlations can hold clinical relevance, despite requiring critical thinking and further study. Possibly, a weak correlation may indicate a subtle but consistent relationship that, when combined with other clinical indicators, may expand knowledge about patient management. Furthermore, weak associations provide information about trends or future hypothesis generation and support approaches to treatment rather than establishing direct causal relationships.

Last but not least, though HF patients with mental disorders or cognitive impairment were excluded from the present study, this exclusion may have shown different results regarding the associations between echocardiography use and self-care. Omission of this group of patients potentially limits the applicability of the findings to the broader HF population. Other confounding factors include education level (affects self-management skills), socioeconomic status (impacts access to medications, diet), comorbidity and polypharmacy (reduce adherence and worsen symptoms), and social support (caregiver involvement improves adherence).

Limitations of the study

In cross-sectional studies, it is not possible to determine the direction or establish causality of observed associations. A longitudinal design (repeated measurements at 6-12 months) can assess changes in self-care and associated factors. Furthermore, the non-random sampling (convenience sampling) of the present study is not representative of all patients with HF in Greece, limiting the generalizability of the findings. Also, in a larger sample size, it is possible that other associations would achieve statistical significance. Self-care was assessed through self-report, which is an additional issue of systematic bias, as it may not reflect actual behavior. The interview method employed does not preclude the potential for researcher bias, which may be manifested through the phrasing of questions, non-verbal cues, or the researcher's expectations. Such biases can influence participants' responses, potentially directing them toward predetermined outcomes (researcher bias). Also, the echocardiography measurements were made by the cardiologist, which means that an additional limitation may be the observer bias (observer effect). The study was conducted exclusively in a single center in the prefecture of Attica, and the findings may not reflect the diversity of other clinical settings, due to the characteristics or protocols of the specific center. The possibility of a center-related effect on the self-care of patients with HF (center effect) may affect the generalizability of the results. Finally, the discussion of the results may have been influenced by the search strategy, including studies published in English and Greek and electronic databases. 

## Conclusions

HF outpatients had moderate levels of self-care. The higher the LVEF and the self-rated QoL, the better the patients' self-care, whereas the higher the E/e' ratio, the IVC diameter, the LA diameter, and the self-rates of dyspnea and change in activities, the poorer the patients' self-care. 

It was found that an increase of one unit in the IVC index and the QoL score was associated with a decrease in self-care scores on the EHFScBS by 0.36 and 0.78 points, respectively.

Objective findings from echocardiography may increase patients' awareness of the disease, thereby enhancing motivation to adhere to recommended self-care practices. Moreover, echocardiographic results allow clinicians to tailor interventions to the individual's health status, guide fluid and sodium management, and provide measurable feedback on disease progression, reinforcing the importance of sustained self-care.

## Appendices

**Table 6:** The nine-item European Heart Failure Self-care Behaviour Scale Table Source: Jaarsma et al. [9]. The scale is freely available on the internet, and no license is needed (https://liu.se/en/research/european-heart-failure-self-care-behaviour-scale).

Items	I completely agree				I don't agree at all
I weigh myself every day	1	2	3	4	5
If my shortness of breath increases, I contact my doctor or nurse	1	2	3	4	5
If my feet/legs become more swollen than usual, I contact my doctor or nurse	1	2	3	4	5
If I gain 2 kg in one week, I contact my doctor or nurse	1	2	3	4	5
I limit the amount of fluids I drink (not more than 1.5-2 liters/day)	1	2	3	4	5
If I experience increased fatigue, I contact my doctor or nurse	1	2	3	4	5
I eat a low-salt diet	1	2	3	4	5
I take my medication as prescribed	1	2	3	4	5
I exercise regularly	1	2	3	4	5

## References

[1] Aghajanloo A, Negarandeh R, Janani L, Tanha K, Hoseini-Esfidarjani SS (2021). Self-care status in patients with heart failure: systematic review and meta-analysis. Nurs Open.

[2] D'Souza PJ, George LS, Paramasivam G, Devasia T, George A, Nayak BS, Kusumavathi P (2024). Knowledge and self-care behavior among heart failure patients in South India. J Educ Health Promot.

[3] Riegel B, De Maria M, Barbaranelli C (2022). Symptom recognition as a mediator in the self-care of chronic illness. Front Public Health.

[4] Chen YY, Borkowski P, Nazarenko N (2024). Demographic and clinical characteristics of New York City Health + Hospitals HIV Heart Failure (NYC4H cohort): cohort profile. BMJ Open.

[5] Peters AE, Clare RM, Chiswell K, Felker GM, Kelsey A, Mentz R, DeVore AD (2023). Echocardiographic features beyond ejection fraction and associated outcomes in patients with heart failure with mildly reduced or preserved ejection fraction. Circ Heart Fail.

[6] Senthamil Selvan PK, Pandiyan PR, Prasanth R, Rajendran R, Subramanian Lakshmi UK, Irfan S (2025). Echocardiographic predictors of readmission and mortality in elderly patients with heart failure and preserved ejection fraction: a prospective observational study. Cureus.

[7] Irfan S, Padmakaran V, Prasanth R (2025). Clinical and echocardiographic profile of patients with heart failure with reduced ejection fraction: a retrospective outcome analysis. Cureus.

[8] Wang N, Rueter P, Ng M (2024). Echocardiographic predictors of cardiovascular outcome in heart failure with preserved ejection fraction. Eur J Heart Fail.

[9] Jaarsma T, Arestedt KF, Mårtensson J, Dracup K, Strömberg A (2009). The European Heart Failure Self-care Behaviour Scale revised into a nine-item scale (EHFScB-9): a reliable and valid international instrument. Eur J Heart Fail.

[10] Lambrinou E, Kalogirou F, Lamnisos D (2014). The Greek version of the 9-item European Heart Failure Self-care Behaviour Scale: a multidimensional or a uni-dimensional scale?. Heart Lung.

[11] Sedlar N, Lainscak M, Mårtensson J, Strömberg A, Jaarsma T, Farkas J (2017). Factors related to self-care behaviours in heart failure: a systematic review of European Heart Failure Self-Care Behaviour Scale studies. Eur J Cardiovasc Nurs.

[12] Kim SH, Hwang SY, Shin JH, Lim YH (2021). Self-care and related factors associated with left ventricular systolic function in patients under follow-up after myocardial infarction. Eur J Cardiovasc Nurs.

[13] Lycholip E, Thon Aamodt I, Lie I (2018). The dynamics of self-care in the course of heart failure management: data from the IN TOUCH study. Patient Prefer Adherence.

[14] Pobrotyn P, Mazur G, Kałużna-Oleksy M, Uchmanowicz B, Lomper K (2021). The level of self-care among patients with chronic heart failure. Healthcare (Basel).

[15] Tchernodrinski S, Lucas BP, Athavale A, Candotti C, Margeta B, Katz A, Kumapley R (2015). Inferior vena cava diameter change after intravenous furosemide in patients diagnosed with acute decompensated heart failure. J Clin Ultrasound.

[16] da Conceição AP, dos Santos MA, dos Santos B (2015). Self-care in heart failure patients. Rev Lat Am Enfermagem.

[17] Li J, Wang C, Dong HW, Qi J, Rao C, Li Q, He K (2025). Inferior vena cava diameter in patients with chronic heart failure and chronic kidney disease: a retrospective study. Eur J Med Res.

[18] Jobs A, Brünjes K, Katalinic A, Babaev V, Desch S, Reppel M, Thiele H (2017). Inferior vena cava diameter in acute decompensated heart failure as predictor of all-cause mortality. Heart Vessels.

[19] Lee HF, Hsu LA, Chang CJ, Chan YH, Wang CL, Ho WJ, Chu PH (2014). Prognostic significance of dilated inferior vena cava in advanced decompensated heart failure. Int J Cardiovasc Imaging.

[20] Sampath-Kumar R, Ben-Yehuda O (2023). Inferior vena cava diameter and risk of acute decompensated heart failure rehospitalisations. Open Heart.

[21] Obokata M, Borlaug BA (2018). The strengths and limitations of E/e' in heart failure with preserved ejection fraction. Eur J Heart Fail.

[22] Li Q, Huang H, Lu X (2023). The association between left ventricular end-diastolic diameter and long-term mortality in patients with coronary artery disease. Rev Cardiovasc Med.

[23] Pastore MC, Vigna M, Saglietto A (2025). Prognostic value of left atrial strain in acute and chronic heart failure: a meta-analysis. ESC Heart Fail.

[24] Man DE, Motofelea AC, Buda V (2025). Left atrial strain in patients with chronic heart failure with preserved ejection fraction: a narrative review. Life (Basel).

[25] Tsai MF, Hwang SL, Tsay SL, Wang CL, Tsai FC, Chen CC, Huang TY (2013). Predicting trends in dyspnea and fatigue in heart failure patients' outcomes. Acta Cardiol Sin.

[26] Vicent L, Nuñez Olarte JM, Puente-Maestu L (2017). Degree of dyspnoea at admission and discharge in patients with heart failure and respiratory diseases. BMC Palliat Care.

[27] Johnson MJ, Cockayne S, Currow DC (2019). Oral modified release morphine for breathlessness in chronic heart failure: a randomized placebo-controlled trial. ESC Heart Fail.

[28] Polikandrioti M (2022). Perceived social isolation in heart failure. J Innov Card Rhythm Manag.

[29] Piotrowicz K, Krzesiński P, Galas A (2024). Health-related quality of life and self-care in heart failure patients under telecare-insights from the randomized, prospective, controlled AMULET trial. Front Public Health.

[30] Polikandrioti M (2021). Patient perceptions and quality of life in pacemaker recipients. J Innov Card Rhythm Manag.

[31] Chamsi-Pasha MA, Sengupta PP, Zoghbi WA (2017). Handheld echocardiography: current state and future perspectives. Circulation.

[32] Ware P, Dorai M, Ross HJ, Cafazzo JA, Laporte A, Boodoo C, Seto E (2019). Patient adherence to a mobile phone-based heart failure telemonitoring program: a longitudinal mixed-methods study. JMIR Mhealth Uhealth.

[33] Smith J, Waters S, Campbell B, Chambers J (2017). Communicating echocardiography results to patients: a future role for the clinical scientist?. Echo Res Pract.

